# Exploring potential cytokine profiles as diagnostic biomarkers for brucellosis in Mediterranean Buffaloes

**DOI:** 10.3389/fvets.2025.1583858

**Published:** 2025-05-08

**Authors:** Giulia Franzoni, Federica Signorelli, Anna Donniacuo, Lorena Schiavo, Michele Napoletano, Giovanna De Matteis, Francesco Grandoni, Susanna Zinellu, Vincenzo Bove, Silvia Dei Giudici, Esterina De Carlo, Giorgio Galiero, Francesco Napolitano, Alessandra Martucciello

**Affiliations:** ^1^Department of Animal Health, Istituto Zooprofilattico Sperimentale della Sardegna, Sassari, Italy; ^2^Council for Agricultural Research and Economics (CREA)- Research Centre for Animal Production and Aquaculture, Monterotondo (RM), Italy; ^3^National Reference Centre for Hygiene and Technologies of Mediterranean Buffalo Farming and Productions, Istituto Zooprofilattico Sperimentale del Mezzogiorno, Salerno, Italy

**Keywords:** Brucella, biomarkers, IFN-***γ***, cytokines, Mediterranean Buffaloes

## Abstract

Brucellosis is a zoonotic disease, with an important economic impact on the livestock industry and public health worldwide. Both *Brucella abortus* and *Brucella melitensis* can infect Mediterranean Buffalo (*Bubalus bubalis*), leading to infertility and abortion. In ruminants, the standard diagnostic approach involves two serological tests, the Rose Bengal Test and the Complement Fixation Test, applied in parallel, though their specificity requires improvement. Cytokines play a crucial role in coordinating immune responses through complex networks and can serve as biomarkers for various diseases. This study explored the potential use of cytokines as immunological biomarkers for Brucella infection in Mediterranean Buffalo. For this purpose, we included 18 healthy and 20 Brucella-infected buffaloes in our analysis. Heparinized blood samples were stimulated with the Brucella antigen, with PBS as nil control and PWM as lymphocyte viability control. After 16–24 h, plasma levels of IL-1α, IL-1β, IL-4, IL-6, IL-10, IL-17, IL-36Ra, MIP-1α, MIP-1β, MCP-1, CXCL8, IP-10, IFN-*γ*, TNF, and VEGF-A were measured using multiplex ELISA. Our results showed that infected animals released significantly higher levels of IFN-*γ*, IP-10, MCP-1 in response to Brucella antigen compared to healthy controls. Conversely, healthy animals released instead higher levels of IL-1α, IL-1β, IL-6 and IL-10 following antigen stimulation compared to infected animals. Finally, sequential canonical discriminant analyses were performed to generate predictive cytokine profiles for each group. The findings indicated that a combination of five cytokines (IFN-*γ*, IP-10, IL-1α, IL-1β, IL-6) can effectively distinguished infected from healthy buffaloes. Overall, this study suggests that incorporating these key immune cytokines could improve the diagnostic accuracy of brucellosis in Mediterranean Buffalo.

## 1 Introduction

Brucellosis is a zoonotic disease caused by bacteria belonging to the genus Brucella (1). This disease has significant economic impact for the livestock industry and pose a serious public health concern.

Brucella are Gram-negative coccobacilli and intracellular pathogens capable of surviving and replicating within macrophages (1). Both *Brucella abortus* (*B. abortus*) and *Brucella melitensis* (*B. melitensis*) can infect buffalo species (*Bubalus bubalis*) (2). In Italy, *B. abortus* is the most prevalent Brucella species affecting cattle and buffaloes, with several outbreaks reported in recent years, particularly in the province of Caserta (3). In Mediterranean Buffaloes, the infection leads to infertility and abortion, resulting in considerable economic losses (1). In humans, *B. abortus* infection causes a severe, chronic and debilitating disease (4), primarily transmitted through contaminated milk and raw dairy products (5) or direct contact with infected animals (6).

Italy is currently running a compulsory eradication and surveillance program for brucellosis in cattle and Mediterranean Buffaloes. In regions not yet declared free of the disease, surveillance is conducted through serological testing of all animals over 1 year old (7). To enhance specificity, at least two diagnostic tests should be used in parallel (8).

A widely accepted diagnostic approach combines the Rose Bengal Test (RBT) with the Complement Fixation Test (CFT) (8). Nevertheless, these tests present some limitations, particularly in cattle and buffaloes, due to their low specificity. False-positive serological reactions (FPSR) might result from exposure to cross reacting microorganisms (9). Both RBT and CFT can detect antibodies produced to the S-LPS and false positive results might occur in animals exposed to Gram-negative bacteria with LPS O-chains similar to those of brucellae. These bacteria include *Escherichia coli O:157*, *Salmonella group N* (O:30), and *Yersinia enterocolitica O:9*. Notably, *Y. enterocolitica O:9* is a major cause of FPSR in diagnosis of brucellosis in bovine and buffaloes (9, 10).

The lack of Brucella specific antigen highlights the need to develop new diagnostic methods, in order to improve the efficacy of eradication strategies and to avoid un-necessary animal sacrifices. In addition, in Italy Mediterranean buffaloes are regarded as a national livestock heritage (7, 11). Accurate diagnosis is essential for an effective surveillance in brucellosis-free areas and for achieving the final stages of eradication (11). Advancing our understanding of host immune responses to Brucella is crucial for developing improved diagnostic strategies and ultimately eliminating the disease from affected infected regions.

In our previous studies, we demonstrated the effectiveness of the interferon-gamma (IFN-*γ*) assay test for diagnosing *Mycobacterium bovis* (*M. bovis*) infection in Mediterranean Buffaloes (12, 13). More recently, we identified additional cytokines that could enhance *M. bovis* diagnostic accurac*y* in this species (14). Building on this knowledge, we investigated the potential of key immune cytokines as diagnostic biomarkers for Brucella infection in Mediterranean Buffaloes.

## 2 Materials and methods

### 2.1 Ethical statements

Mediterranean Buffaloes used in this study were analyzed within the context of National and Regional buffalo brucellosis-surveillance program, provided by the EU Delegates Regulations 2020/689 and Campania Region Regional Regulation (DGRC 104/2022) (15, 16).

No animal was harmed or killed for the specific purpose of this study and the experimental procedure was carried out in compliance with the European Directive 210/63/UE and the Italian regulation D Lgs n26/2014.

### 2.2 Animals and study design

Thirty-eight Mediterranean Buffaloes were enrolled in the study and were divided in two groups: healthy (*N* = 18) and infected (*N* = 20).

Infected animals were selected from herds with confirmed brucellosis outbreaks. Infection status was determined according to current legislation using serological tests RBT and CFT, following the guidelines outlined in the WOAH Manual of Diagnostic Tests and Vaccines for Terrestrial Animals (8). RBT is used as a screening test (carried out every 6 months in all herds of Campania region), while CFT is used to confirm RBT positive cases. If an animal tests positive on CFT, the herd is classified as a brucellosis outbreak, then it is subsequently monitored every 21 days using both RBT and CFT (8). Seropositive animals were culled in compliance with national and regional regulations. Brucella presence in selected organs was assessed through PCR and culture isolation (see Section 2.3). Animals included in the infected group were seropositive, with Brucella DNA detected in target organs (Supplementary Table S1). Brucella was isolated from at least one animal per outbreak.

Healthy animals were selected from Officially Brucellosis-Free (OTF) herds in the Campania region (Southern Italy). These animals tested negative during the annual serological screening tests performed in the last 6 years (Supplementary Table S1).

### 2.3 PCR and culture isolation

Lymph nodes (retropharyngeal, supra-mammary, iliac, and mandibular lymph nodes) and other organs (spleen, uterus, mammary gland) were collected and sent to the IZSME (Portici, Italy) for Brucella detection though PCR and culture isolation.

PCR analysis was performed to detect Brucella DNA in the collected tissues. Genomic DNA was extracted using the QIAamp DNA MINI KIT (QIAGEN) following the manufacturer’s instructions, as previously described (17). Then, the identification of the *Brucella* spp. was performed though a real-time PCR assay using TaqMan probes and targeting IS711 (18). The presence of viable Brucella bacteria was assessed through culture isolation, according to the WOAH Manual of Diagnostic Tests and Vaccines for Terrestrial Animals (8). Bacterial isolates were further identified using the VITEK 2 system (3).

### 2.4 Whole blood stimulation and evaluation of cytokines release

Blood samples were collected from the jugular vein and heparin was used as anticoagulant and processed within 8 h of collection.

Whole blood from each animal was dispensed in aliquots of 1 mL, using a 24 well-plate and stimulated with Phosphate-buffered saline (PBS, nil Control Antigen) or Brucella antigen (Brucellergene® OCB) (100 μL per well, corresponding to 1,500–2,500 UI) or Pokeweed Mitogen (PWM, final concentration 1 μg/mL, used as a lymphocyte viability control). After incubation for 16–24 h at 37°C in a humidified atmosphere, plasma samples were collected. Then, levels of 15 key immune cytokines (IFN-*γ*, IL-1α, IL-1β, IL-4, IL-6, IL-10, IL17, IL-36Ra, MIP-1α, MIP-1β, MCP-1, IP-10, CXCL8, TNF, VEGF-A) were measured using Bovine Cytokine/Chemokine Magnetic Bead Panel Multiplex assay (Merck Millipore, Darmstadt, Germany) and a Bioplex MAGPIX Multiplex Reader (Bio-Rad, Hercules, CA, United States), as previously described (14). All samples were tested in duplicate (two technical replicates).

Antigen*-*specific cytokine in responses were calculated by subtracting baseline cytokines concentrations (PBS, nil control) from those of the antigen condition (Brucellergene® OCB).

### 2.5 Statistical analysis

Levels of the 15 tested cytokines were analyzed using the general linear model (GLM) to estimate the mean of each trait per stimulus (PBS, BRC, and PWM) within groups (healthy and infected):


$$Y_{jk}=\mu +G_{j}+e_{jk}$$

where Y_jk_ is the trait measured for each animal, μ is the overall mean, G_j_ is the fixed effect of the stimuli (j = 3 levels: PBS, BRC, and PWM), and e_jk_ is the random residual effect of each observation.

The statistical significance of all traits and least-square means were assessed by Dunnet’s multiple test in the GLM procedure.

Additionally, the difference (∆_cytokine) between the level of each specific cytokine measured in the Brucella antigen condition (BRC) and its baseline concentration (PBS) was analyzed and displayed by GraphPad Prism 10.01 (GraphPad Software Inc., La Jolla, CA, United States).

A multivariate approach was conducted using canonical discriminant analysis (CDA) on 15, 7 and finally 5 ∆_cytokines by the CANDISC Procedure.

The significance level for both statistical analyses was set at a *p-*value < 0.05.

The CDAs were conducted by categorizing animals prior to healthy and infected. The CANDISC method was utilized to estimate linear functions of all quantitative variables that best discriminated against the groups while minimizing the variation within each group.

All statistical analyses were performed with SAS software version 9.4.

## 3 Results

Whole blood samples from healthy (*N* = 18) and infected (*N* = 20) Mediterranean Buffaloes were stimulated with a specific Brucella antigen (Brucellergene® OCB), alongside controls, and then the release of key immune cytokines was determined.

In both infected and healthy animal groups, PWM triggered the release of IFN-*γ*, IL-17, IL-10, and TNF indicating that T cells viability and functionality were not altered by treatments that could have affected the analyses (e.g., corticosteroid administration) (Table 1). PWM triggered also the release of chemokines IP-10, MIP-1α, and MIP-1β (Table 1), suggesting that the viability of other immune cells (e.g., monocytes) was not altered as well.

**Table 1:** Cytokine production in whole blood from healthy and Brucella-infected Mediterranean Buffaloes.

	PBS	BRC	PWM	PBS-BRC	PBS-PWM
	LSM ± SE	LSM ± SE	LSM ± SE	*p*-value	*p*-value
Healthy					
Cytokines					
IFN-γ	6 ± 102	11 ± 102	1,158 ± 102	0.9993	**0.0001**
IL-17	9 ± 149	35 ± 149	1,106 ± 149	0.9887	**0.0001**
IP-10	1,611 ± 179	1,692 ± 179	3,721 ± 179	0.9887	**0.0001**
IL-6	1,085 ± 210	1,430 ± 210	1,436 ± 210	0.4109	0.3984
TNF	1,772 ± 604	3,943 ± 604	5,142 ± 604	**0.0261**	**0.0005**
IL-1ɑ	34 ± 13	70 ± 13	91 ± 13	0.1097	**0.0069**
IL-1β	321 ± 295	2,218 ± 295	891 ± 295	**0.0001**	0.2992
IL-4	90 ± 26	89 ± 26	269 ± 26	0.9995	**0.0001**
CXCL8	3,628 ± 355	3,451 ± 366	4,122 ± 346	0.9159	0.5091
IL-10	380 ± 148	469 ± 148	1,819 ± 148	0.8771	**0.0001**
MIP-1ɑ	2,328 ± 481	3,914 ± 481	5,506 ± 481	**0.0434**	**0.0001**
MIP-1β	322 ± 60	582 ± 60	871 ± 60	**0.0064**	**0.0001**
IL-36Ra	480 ± 55	452 ± 55	467 ± 55	0.9074	0.9781
VEGF	188 ± 19	233 ± 19	140 ± 19	0.1827	0.1475
MCP-1	5,075 ± 175	4,602 ± 175	5,212 ± 175	0.1092	0.8036
Infected					
Cytokines					
IFN-γ	16 ± 120	861 ± 120	1,133 ± 120	**0.0001**	**0.0001**
IL-17	2 ± 51	23 ± 51	364 ± 51	0.9429	**0.0001**
IP-10	2,111 ± 246	3,093 ± 246	3,109 ± 246	**0.0126**	**0.0111**
IL-6	1,316 ± 187	1,005 ± 187	1,159 ± 187	0.3957	0.7768
TNF	2,163 ± 718	2,790 ± 718	5,860 ± 718	0.7625	**0.0011**
IL-1ɑ	44 ± 25	52 ± 25	156 ± 25	0.9608	**0.0055**
IL-1β	291 ± 175	755 ± 175	1,702 ± 175	0.1193	**0.0001**
IL-4	82 ± 18	85 ± 18	115 ± 18	0.9867	0.3391
CXCL8	3,351 ± 312	3,412 ± 321	3,193 ± 331	0.9868	0.9165
IL-10	378 ± 99	334 ± 99	1,311 ± 99	0.9337	**0.0001**
MIP-1ɑ	2,553 ± 411	3,494 ± 411	5,000 ± 411	0.1929	**0.0002**
MIP-1β	466 ± 76	727 ± 76	946 ± 76	**0.0348**	**0.0001**
IL-36Ra	297 ± 25	296 ± 25	292 ± 25	0.9997	0.9867
VEGF	194 ± 23	224 ± 23	177 ± 23	0.5691	0.8385
MCP-1	4,964 ± 173	4,914 ± 173	5,268 ± 173	0.9704	0.3634

Whole blood samples were stimulated with PBS (nil control) or Brucellergene® OCB antigen (BRC), or PWM (positive control). The concentration of 15 cytokines were measured using multiplex ELISA. The table presents the Least Squares Mean (LSM), Standard Estimated Error (SEE), and statistical differences (*p*-value) between conditions. *p*-value < 0.05 were considered statistically significant and are marked in bold.

In the infected group, but not in the healthy group, stimulation with Brucella antigen (BRC) led to a significantly higher release of IFN-*γ* and IP-10 compared to the nil control (Table 1). Both groups exhibited an increase in MIP-1β levels in response to Brucella antigen. In the healthy group, additional pro-inflammatory cytokines (TNF, IL-1β, MIP-1α) were also released upon antigen stimulation, suggesting an innate immune response to Brucella components in naïve animals.

Then, differences between the two groups (healthy and infected) in terms of antigen-specific cytokine releases were assessed. Baseline cytokines levels (PBS, nil control) were subtracted from those in the antigen condition (Brucellergene® OCB) and then differences between groups were analyzed.

**Figure 1:**
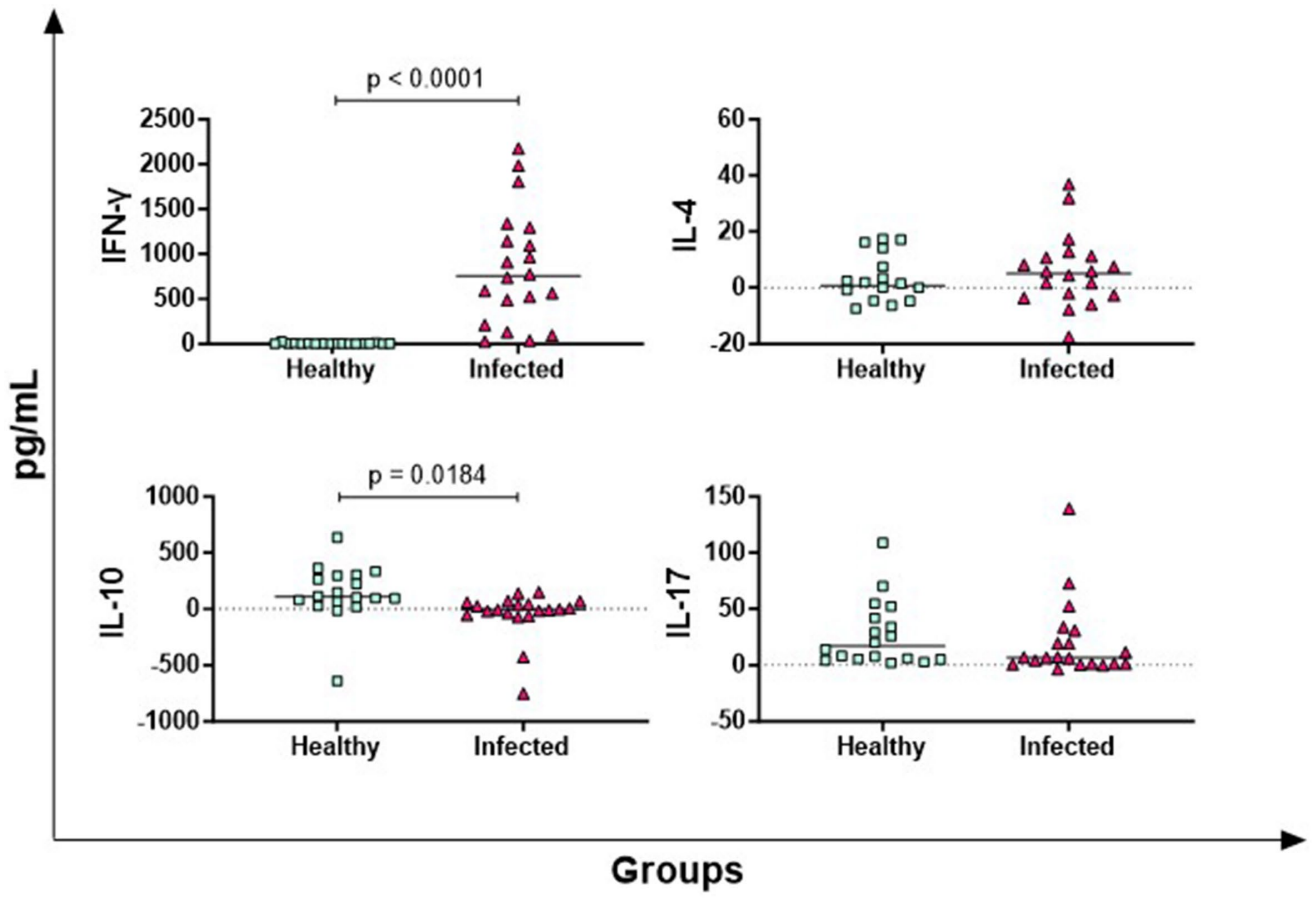
Antigen-specific release of T-cell cytokines in Brucella-Infected buffaloes. Blood samples from Brucella-infected (*n* = 20) and healthy (*n* = 18) Mediterranean Buffaloes were collected using heparin as anticoagulant. Whole blood was stimulated with PBS (nil control) or Brucellergene® OCB antigen (BRC). After 16–24 h of incubation, plasma was collected, and levels of T-cell cytokines (IFN-γ, IL-17, IL-4, IL-10) were determined using multiplex ELISA. Brucella-specific cytokines values were calculated by subtracting baseline cytokines levels (PBS) from those in antigen condition. Differences between groups are shown, with statistical significance set at *p* < 0.05.

In Figure 1, the results of four key T cell cytokines are presented: IFN-*γ* (Th1 response marker), IL-4 (Th2 response marker), IL-17 (mainly released by Th17), IL-10 (Treg-associated immunosuppressive cytokine) (19). Infected animals released higher levels of antigen-specific IFN-γ compared to healthy animals (*p* < 0.0001), whereas no significant differences were observed for IL-4 and IL-17. Conversely, healthy animals produced higher antigen-specific IL-10 levels compared to -infected subjects (*p* = 0.0184) (Figure 1).

**Figure 2:**
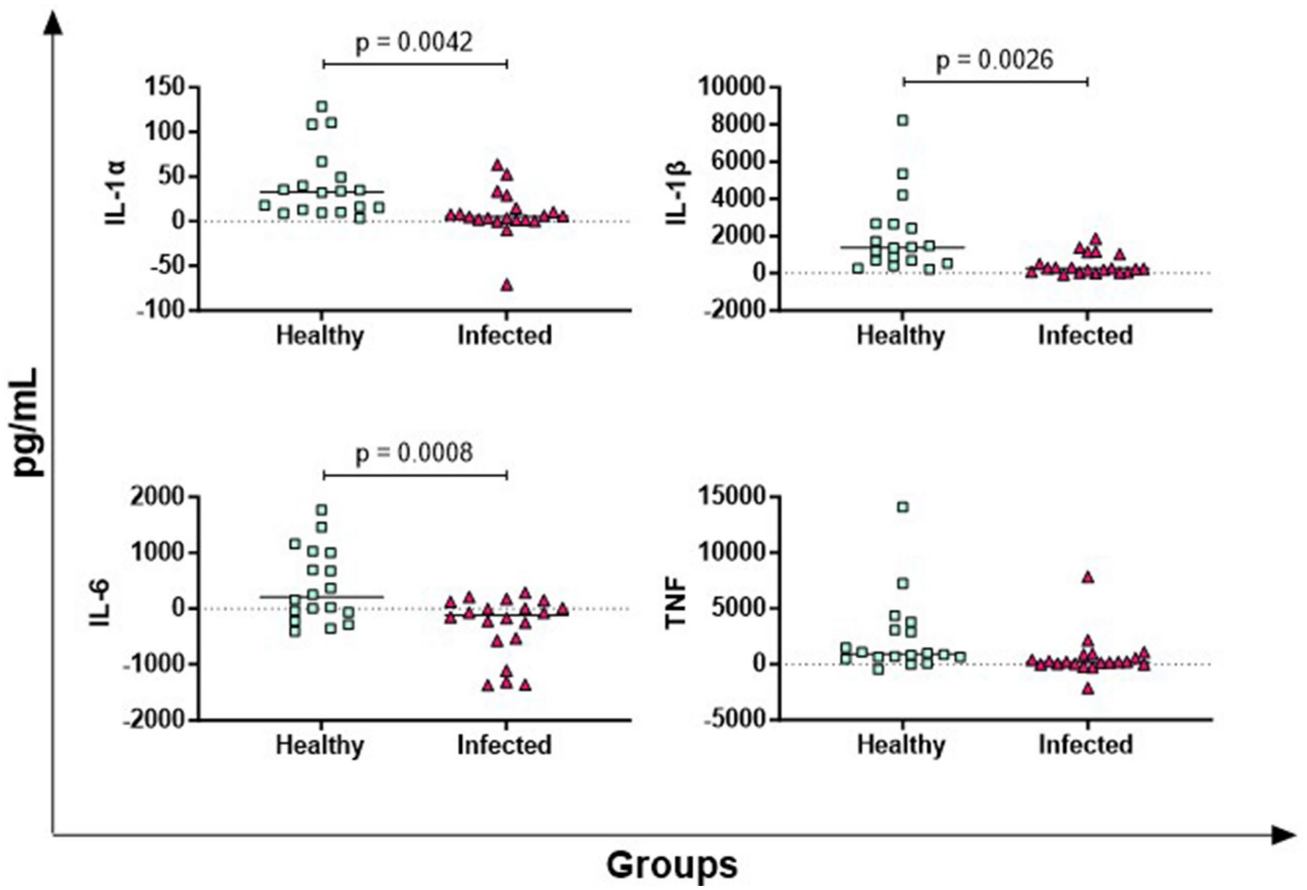
Antigen-specific release of pro-inflammatory cytokines in Brucella-infected buffaloes. Blood samples from Brucella-infected (*n* = 20) and healthy (*n* = 18) Mediterranean Buffaloes were collected using heparin as anticoagulant. Whole blood was stimulated with PBS (nil control) or Brucellergene® OCB antigen (BRC). After 16–24 h of incubation, plasma was collected, and levels of pro-inflammatory cytokines (IL-1α, IL-1β, IL-6, TNF) were measured using multiplex ELISA. Brucella-specific cytokines values were determined by subtracting baseline cytokines levels (PBS) from those in the antigen condition. Differences between the groups are shown, with statistical significance set at *p* < 0.05.

In Figure 2, the results of four key pro-inflammatory cytokines are shown: IL-1α, IL-1β, IL-6, TNF (19). Infected animals released lower levels of antigen-specific IL-1α, IL-1β, IL-6 compared to healthy subjects, whereas TNF levels did not differ significantly between groups (Figure 2).

**Figure 3:**
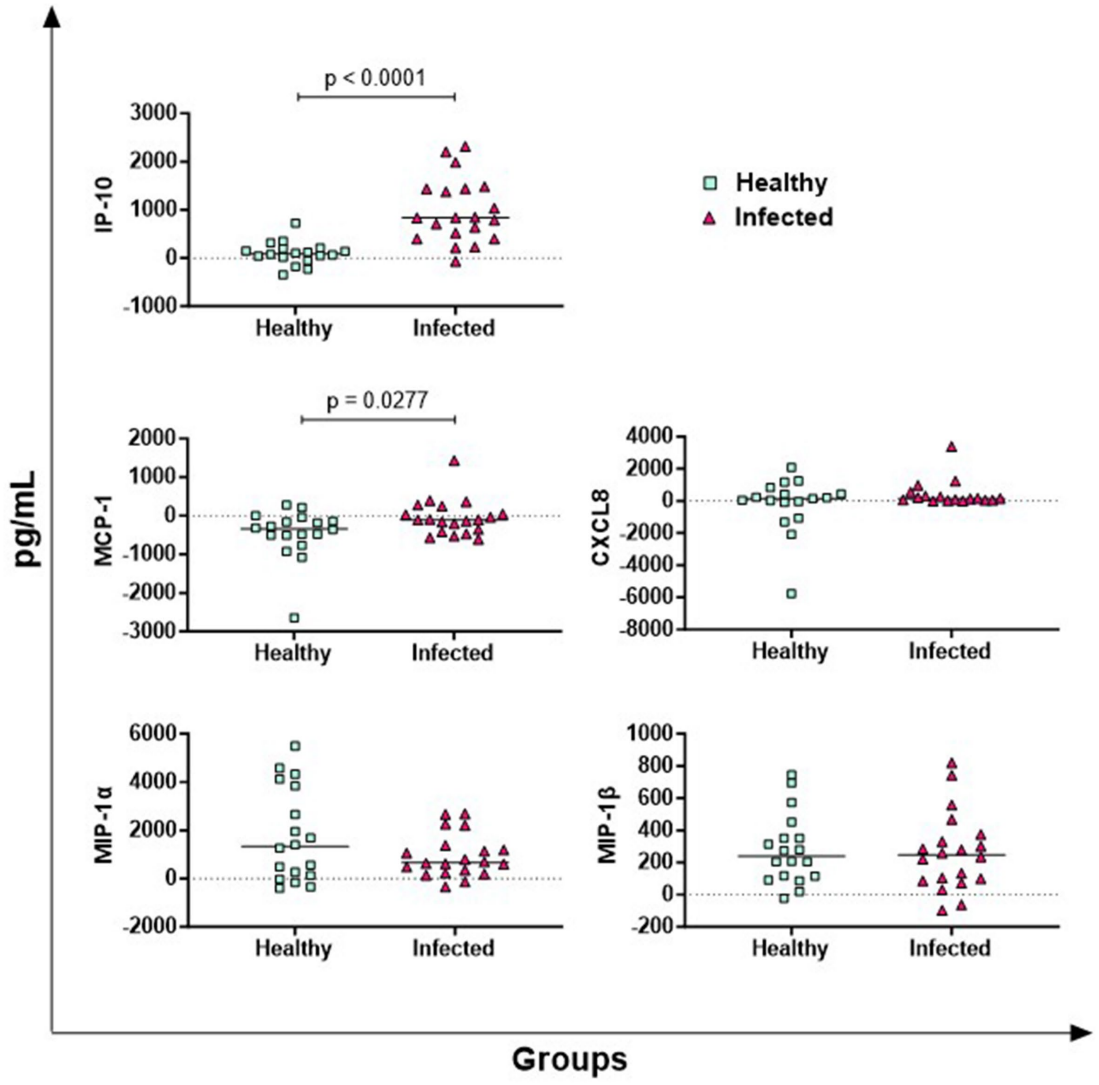
Antigen-specific release of chemokines in Brucella-Infected buffaloes. Blood samples from Brucella-infected (*n* = 20) and healthy (*n* = 18) Mediterranean Buffaloes were collected using heparin as anticoagulant. Whole blood was stimulated with PBS (nil control) or antigen Brucellergene® OCB antigen (BRC). After 16–24 h of incubation, plasma was collected, and levels of key chemokines (IP-10, MIP-1α, MIP-1β, MCP-1, CXCL8) were measured using multiplex ELISA. Brucella-specific cytokines values were calculated by subtracting baseline cytokines levels (PBS) from those in antigen condition. Differences between the groups are shown, with statistical significance set at *p* < 0.05.

The results of five key chemokines are presented in Figure 3. No differences between groups were observed for MIP-1α and MIP-1β, whereas infected animals released higher levels of antigen-specific IP-10 (*p* < 0.0001) and MCP-1 (*p* = 0.0277) compared to healthy subjects. Regarding CXCL8, 5 out of 38 buffaloes presented cytokine levels higher than the detection limit of the kit, so they were excluded from the analysis. In the other animals (17 infected vs. 16 healthy), no differences were observed between groups (Figure 3).

**Figure 4:**
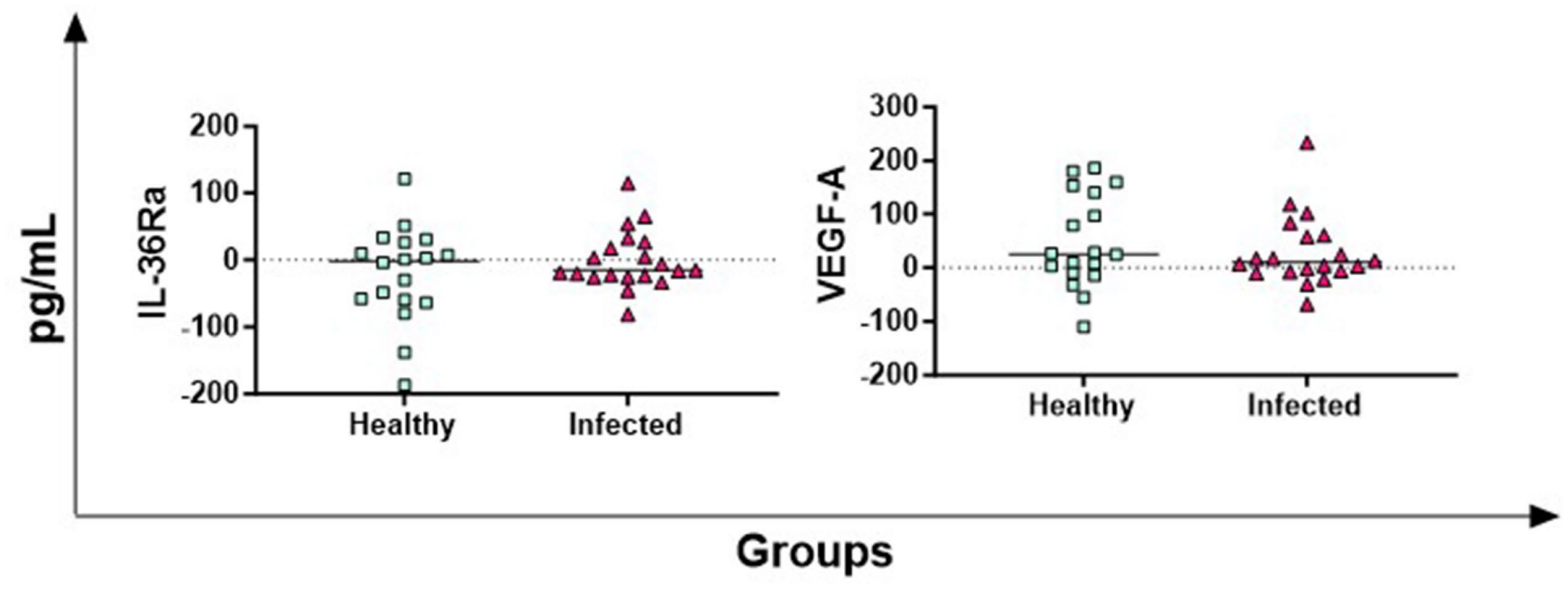
Antigen-specific release of IL-36Ra and VEGF-A in Brucella-Infected buffaloes. Blood samples from Brucella-infected (*n* = 20) and healthy (*n* = 18) Mediterranean Buffaloes were collected using heparin as anticoagulant. Whole blood was stimulated with PBS (nil control) or Brucellergene® OCB antigen (BRC). After 16–24 h of incubation, plasma was collected, and levels of IL-36Ra and VEGF-A were determined through multiplex ELISA. Brucella-specific cytokines values were quantified by subtracting baseline cytokines levels (PBS) from those in the antigen condition. Differences between the groups are shown, with statistical significance set at *p* < 0.05.

The antigen-specific release of IL-36Ra (receptor antagonist) and VEGF-A (growth factor) was also investigated, but no significant differences between groups were observed (Figure 4).

**Figure 5:**
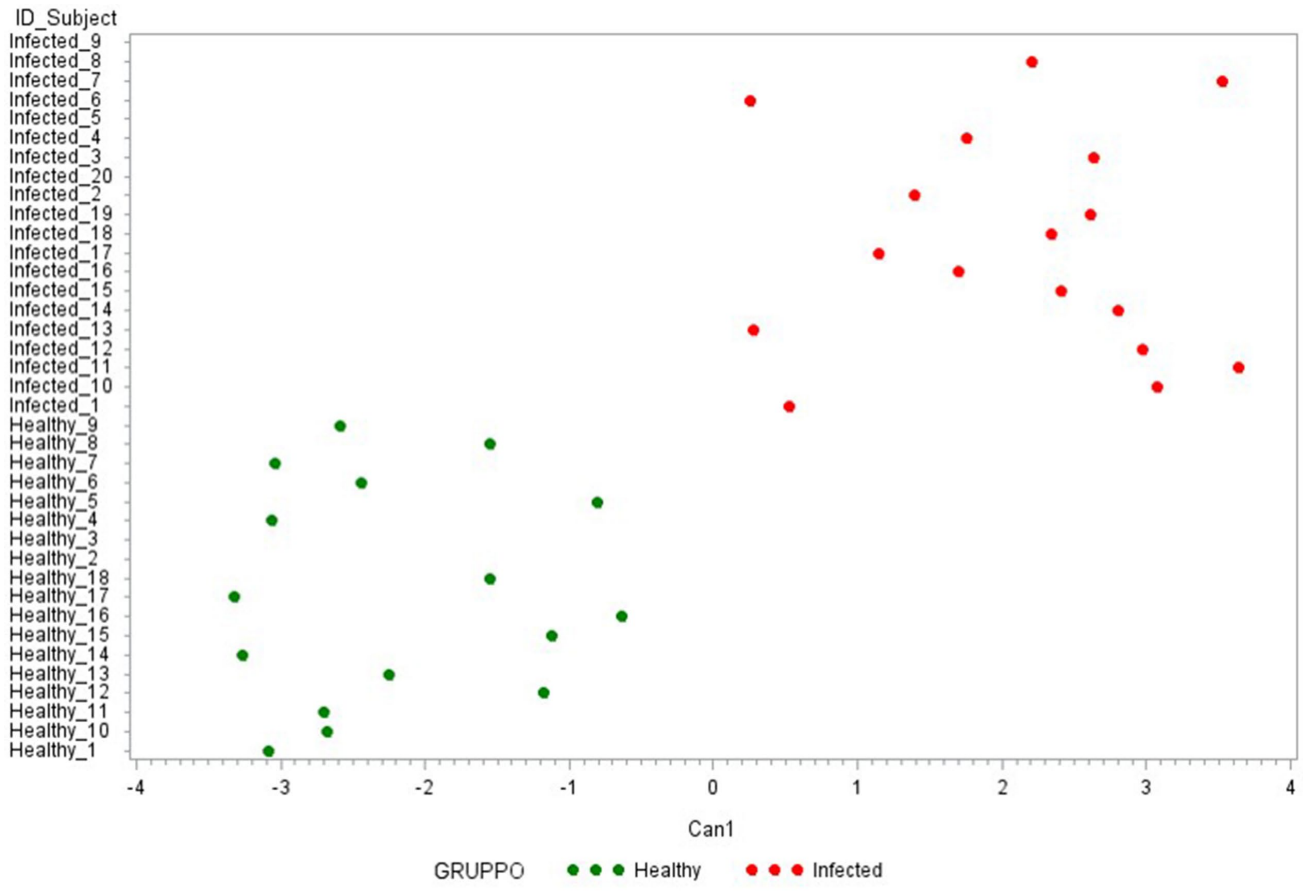
Plot from canonical discriminant analysis. Canonical discriminant analysis on 15 Δ_cytokines by the CANDISC Procedure. Animals belonging to the two groups are displayed based on the canonical function Can1.

Canonical discriminant analysis was then used to generate predictive cytokine profiles distinguishing healthy and infected animals as potential diagnostic biomarkers. First, a CDA was performed with the 15 cytokines analyzed in the study. As presented in Figure 5, these 15 cytokines can clearly differentiate between the two groups under evaluation. Table 2A reports the factor loading (FL) for each cytokine in the canonical variable (Can 1), showing a positive and high correlation with ∆_IFN-*γ*, ∆_IP-10 (FL ≥ 0.70), characterizing the infected group, and a negative correlation with ∆_IL-6, ∆_IL-10, ∆_IL1-*β*, and ∆_IL1-*α* (FL ≥ 0.50), characterizing the healthy one.

**Table 2:** Correlations between canonical function (Can 1) and original variables.

A	
∆_cytokine	Can 1
**∆_IFN-γ**	**0.74**
**∆_IP-10**	**0.73**
**∆_IL-6**	**−0.59**
**∆_IL-10**	**−0.59**
**∆_IL-1β**	**−0.56**
**∆_IL-1α**	**−0.50**
∆_MCP1	0.44
∆_TNF	−0.36
∆_MIP-1α	−0.28
∆_CXCL8	0.23
∆_IL-36Ra	0.19
∆_VEGF	−0.18
∆_IL4	0.11
∆_IL-17	−0.03
∆_MIP-1β	−0.02

B	
∆_cytokine	Can 1
**∆_IFN-γ**	**0.8**
**∆_IP-10**	**0.79**
**∆_IL-6**	**−0.61**
**∆_IL-1β**	**−0.56**
**∆_IL-1α**	**−0.54**
∆_IL-10	−0.45
∆_MCP1	0.42

C	
∆_cytokine	Can 1
**∆_IFN-γ**	**0.82**
**∆_IP-10**	**0.80**
**∆_IL-6**	**−0.63**
**∆_IL-1β**	**−0.58**
**∆_IL-1α**	**−0.55**

The difference (∆_cytokine) between the level of each specific cytokine measured in the antigen condition (Brucellergene® OCB) and its baseline cytokine concentration (PBS), and their weight in the Can 1, are shown: A—the 15 tested cytokines. B—the seven cytokines that significantly differentiate the healthy and infected groups. C—the five cytokines with a FL ≥ 0.50 in the previous Can 1 (B). The heavier correlation coefficients are marked in bold.

**Figure 6:**
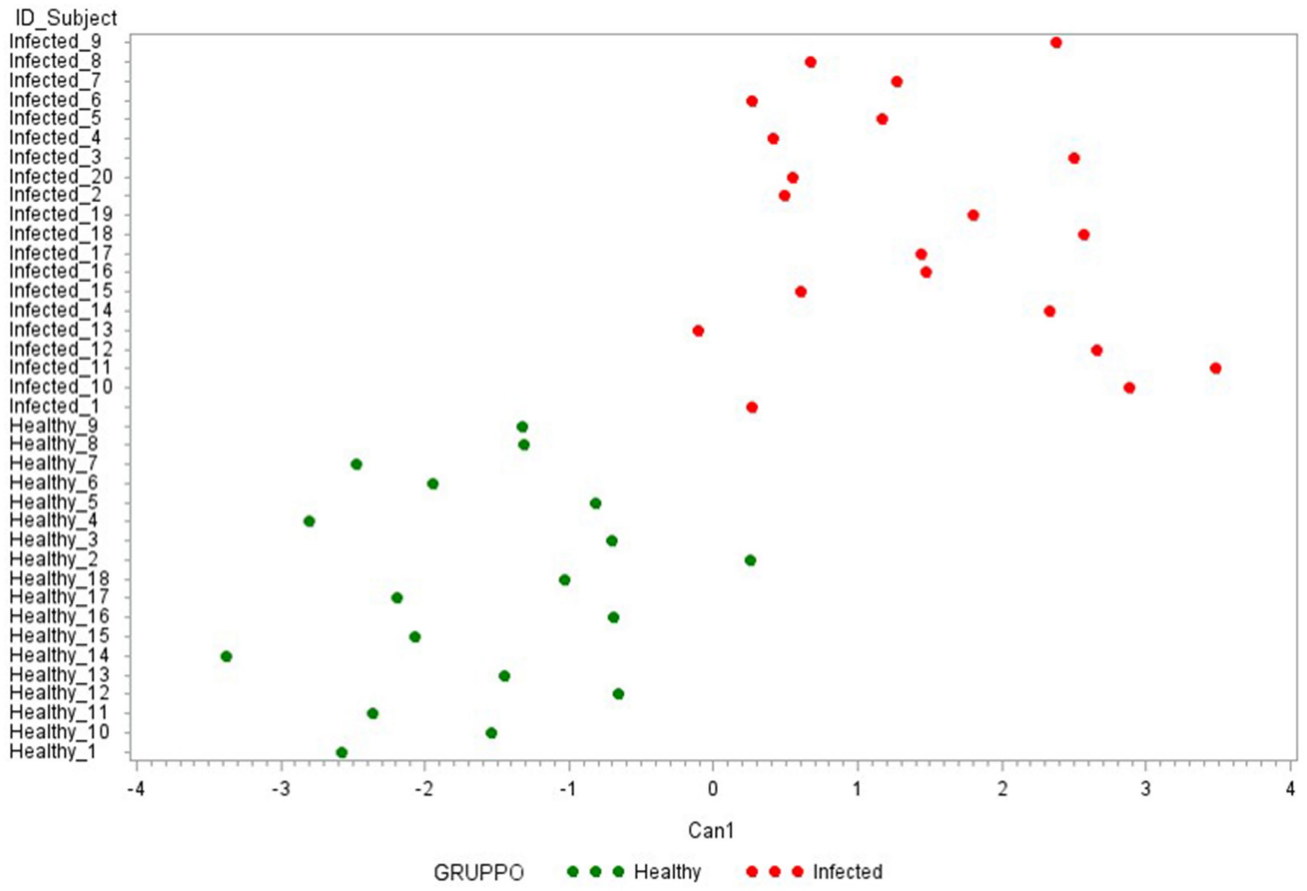
Plot from canonical discriminant analysis. Canonical discriminant analysis on 7 Δ_cytokines by the CANDISC Procedure. Animals belonging to the two groups are displayed based on the canonical function Can1.

Then, to identify the most discriminating combination of cytokines, two other CDAs were conducted. The first one was performed with the 7 ∆_cytokine levels that significantly differentiate the two groups (IFN-*γ*, IP-10, MCP-1, IL-6, IL1-*β*, IL-1*α*, IL-10). As presented in Figure 6, this model clearly discriminated against the two groups, except of one healthy animal (Healthy_2). Table 2B reports the cytokine FL in this new canonical function, revealing a high correlation with ∆_IFN-γ, ∆_IP-10 (0.80–0.79, respectively) and a medium one with IL-6, ∆_IL1-β, and ∆_IL1-α (FL ≥ 0.50).

**Figure 7:**
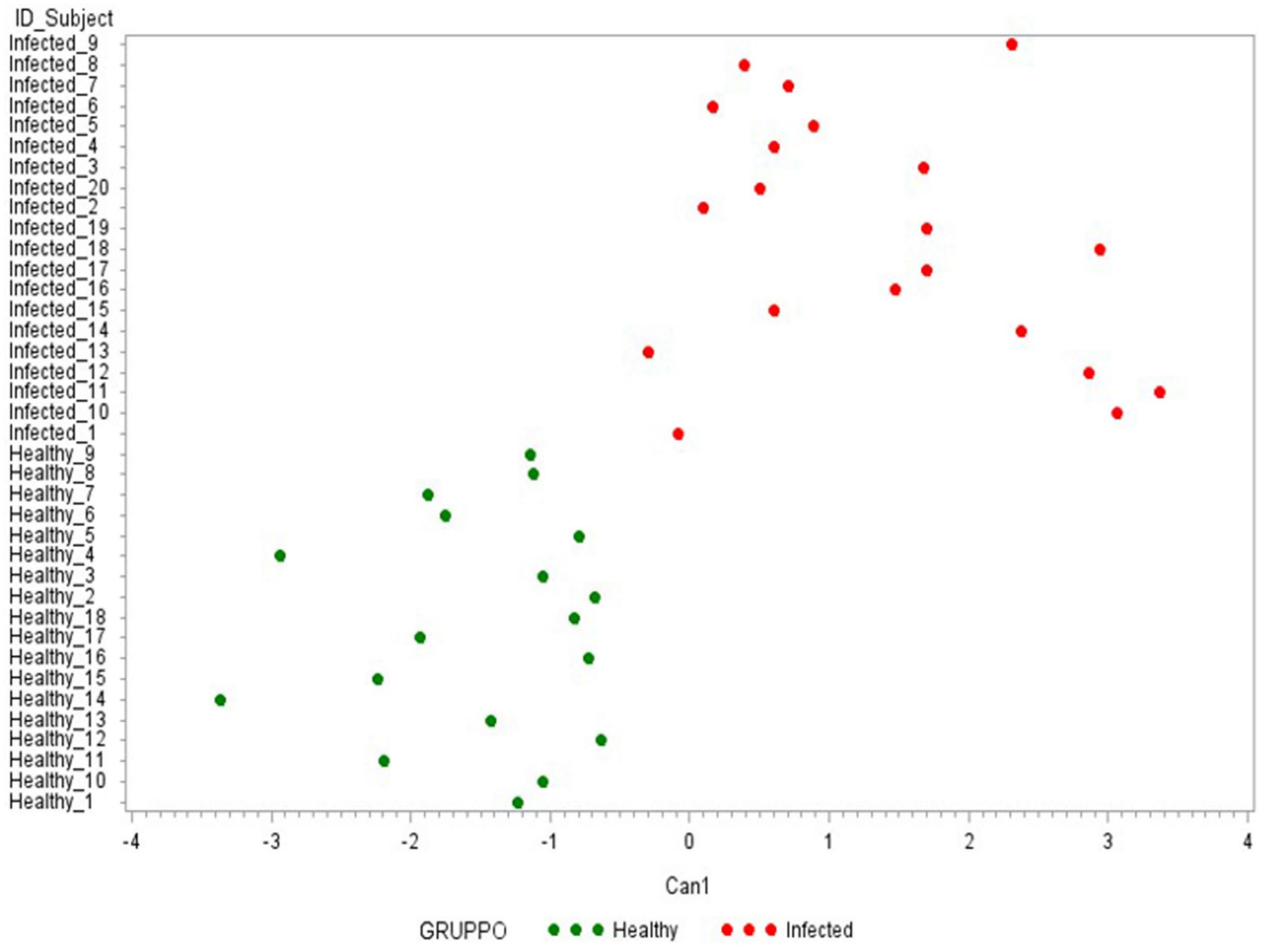
Plot from canonical discriminant analysis. Canonical discriminant analysis on 5 Δ_cytokines by the CANDISC Procedure. Animals belonging to the two groups are displayed based on the canonical function Can1.

Finally, the third CDA (Table 2C) was conducted using only the 5 cytokines with an FL ≥ 0.50 in the previous Can 1: IFN-γ, IP-10, IL-6, IL1-β, and IL1-α. As presented in Figure 7, the use of these 5 cytokines can clearly differentiate the two groups under evaluation (healthy-infected).

## 4 Discussion

Brucella is a globally distributed zoonotic pathogen that affects various domestic and wild animals species, including Mediterranean Buffalo (3, 20). Early and accurate detection of Brucella infection in this specie is crucial for effective disease control.

A widely accepted diagnostic approach for Brucella in cattle and buffaloes combines two serological assays (the RBT with the CFT), although these test present some disadvantages, such as low specificity. False-positive serological reactions (FPSR) might result from exposure to cross reacting microorganisms, especially Gram-negative bacteria with LPS O-chains similar to those of Brucella (8, 9). There is a need to develop new diagnostic methods, in order to improve the efficacy of eradication strategies and to avoid unnecessary animal sacrifices. Brucella is an intracellular pathogen, which primarily targets macrophages and dendritic cells, employing sophisticated mechanism to both innate and adaptive immune response, enabling its survival and persistence within host cells (20). Resistance to intracellular bacterial pathogens relies on cell-mediated immunity, and it was reported that an adequate Th1 immune response is critical for the clearance of Brucella infection. In humans, IFN-*γ* is the key cytokine involved in the immune response against Brucella (20, 21). Cytokines are crucial mediators of immune responses and their quantification provides insights into physiological and pathological processes, aiding diagnosis and treatment, thus they have been widely studied as biomarkers for many diseases. Cytokine testing has the potential to support diagnosis also due to its lack of invasiveness and relative low cost (22).

Biomarker development is a multistep process that starts with its discovery in a pathophysiological context and progresses through various validation phases. This study provides preliminary insights into the role of key immune cytokines as biomarkers for Brucella infection in Mediterranean Buffaloes, with the aim to implement the diagnosis of Brucella in this specie. Understating cytokine responses to antigen-stimulation could offer also a more comprehensive picture of host immunity against this pathogen.

IFN-*γ* is an antiviral cytokine released mainly by NK and activated T cells; it is regarded as a hallmark of a Th1 response, which is associated with resistance to intracellular pathogens (23, 24). Our finding indicates that Brucella-infected buffaloes release higher levels of antigen-specific IFN-γ compared to uninfected controls. Similar observations have been reported in cattle infected with *B. abortus*, where infected animals exhibited elevated IFN-γ levels upon antigen stimulation (25, 26). Likewise, in *B. melitensis* infected sheep, increased IFN-γ secretion was noted following antigen exposure (27, 28). Overall, our data suggest that antigen-specific IFN-γ release could serve as a potential diagnostic marker for Brucella infection in Mediterranean Buffaloes.

We recently observed that Mediterranean Buffaloes infected with *B. abortus* presented lower levels of circulating T and B cells compared to healthy controls (29) and this might result in a different T-cell cytokine pattern in response to antigen stimulation. IL-17 and IL-4 releases were therefore investigated. IL-17 and IL-14 are regarded as hallmark of the Th17 and Th2 response, respectively (19, 30). IL-17 promotes inflammation and a granulocyte-dependent response to pathogens; it triggers the release of pro-inflammatory cytokines and enhances the recruitment of neutrophils (30). IL-4 promotes instead tissue regeneration and the differentiation of Th2 cells (19). Despite the important role of these cytokines in the immune response to several pathogens, little is known about their function during Brucella infection. In humans, the IL-17 serum levels were significantly higher in subjects with brucellosis compared to healthy controls and the levels of this cytokine decreased in patients after specific treatment (31). However, in our study, no differences in antigen-specific IL-17 or IL-4 secretion were observed between infected and healthy buffaloes, suggesting that these cytokines may not be reliable diagnostic markers for brucellosis in this specie.

IL-10 is a cytokine with strong anti-inflammatory action, with the ability to reduce or terminate inflammation (32). It promotes the expansion and persistence of T reg cells, which prevent autoimmunity and limit chronic inflammatory diseases (33). Our data revealed that infected buffaloes release lower levels of antigen-specific IL-10 compared to healthy subjects. Similarly, we observed that Mediterranean Buffaloes with brucellosis release lower levels of three pro-inflammatory cytokines than healthy controls: IL-1α, IL-1β, IL-6. These cytokines are typically released early during infection to initiate inflammation, with IL-10 acting to balance their effects (19, 32). Comparable findings have been reported in human patients with brucellosis, where monocytes exhibited diminished IL-1β, IL-6, and IL-10 secretion in response to LPS stimulation (34). Brucella employs immune evasion strategies, including inflammasome downregulation and inhibition of macrophage polarization toward a pro-inflammatory phenotype (M1) (34, 35). Our results suggest that Brucella infection impairs monocyte ability to release pro-inflammatory cytokines in response to pathogen associated molecular patterns, such as those contained in the antigen Brucellergene® OCB (protein extract of *B. melitensis* strain BB15).

TNF is another pro-inflammatory cytokine, which triggers the release of pro-inflammatory chemokines, effectively recruiting leukocytes to the inflammatory site (36). Studies in humans reported that monocytes and dendritic cells from patients with brucellosis released lower levels of TNF in response to external stimuli compared to healthy controls (34, 37). However, in our study, no differences in antigen-specific TNF secretion were observed between infected and healthy buffaloes. This discrepancy could be attributed to the involvement of other immune cells, such as CD4^+^ and CD8^+^ T cells, in TNF production (38), equalizing the difference between groups in terms of total TNF-release in response to antigen-stimulation. Our previous research suggested that TNF might be a useful biomarker for identifying buffaloes infected with *M. bovis* (14), but the current data indicate that TNF is not a suitable biomarker for Brucella infection.

We also examined the antigen-specific secretion of key chemokines: IP-10, MCP-1, MIP-1α, MIP-1β, CXCL8. Chemokines play a crucial role in immune cell recruitment to infected tissues (39). IP-10 is secreted by monocytes, macrophages, endothelial cells and fibroblasts and its release is sharply enhanced by IFN-*γ* (40). This chemokine triggers the recruitment of monocytes, macrophages, NK cells, and activated T cells to the inflammatory site (40). It has been identified as a promising biomarker for *M. bovis* infection in cattle and buffaloes (14, 41–44). In this study, we observed that Brucella-infected animals release high levels of antigen-specific IP-10 compared to uninfected controls. These data are in agreement with what observed in a mouse models of brucellosis: immune cells of mice immunized with a *B. melitensis* attenuated strains (WR201) release high levels of antigen-specific IP-10 compared to healthy controls (45). Overall, our preliminary data suggest that this chemokine could be a promising biomarker of brucellosis in buffaloes. MCP-1 is a strong chemoattractant for monocytes and it is released mainly by monocytes and macrophages (46). In a mouse model of brucellosis, immune cells of Brucella-infected mice release high levels of antigen-specific MCP-1 compared to healthy controls (45). In agreement, in our study we observed that Brucella-infected animals release high levels of antigen-specific MCP-1 compared to uninfected controls, suggesting that this chemokine could be a promising biomarker of brucellosis in buffaloes. MIP-1α and MIP-1β are pro-inflammatory chemokines mainly produced by monocytes/macrophages (47). They promote recruitment of diverse cell types (chemotaxis of monocytes, dendritic cells, T cells, NK cells, and granulocytes) to the inflammatory sites (47). Previous studies suggest that these chemokines were involved in the immune response to other intracellular bacteria infecting Mediterranean Buffaloes, such as *M. bovis* (14). Our data revealed that infected and healthy buffaloes release similar levels of antigen-specific MIP-1α and MIP-1β, suggesting that these two chemokines could not be useful biomarkers of brucellosis in buffaloes. CXCL8, also known as IL-8, is a strong neutrophil chemoattractant, which triggers the recruitment of neutrophils and other granulocytes to the site of infection, promotes neutrophil degranulation and enhancements in their phagocytic functions (39, 48). This chemokine is released by neutrophils and macrophages in response to Brucella infection (49, 50), but infection results also in a lower ability of macrophages to present antigen to T cells and to release pro-inflammatory cytokines in response to external stimuli (34, 51). Our data revealed that there were no differences between groups, suggesting that the evaluation of CXCL8 will not improve the diagnosis of brucellosis in Mediterranean Buffaloes.

Subsequently, we investigated the antigen-specific release of IL-36Ra and VEGF. IL-36Ra is the receptor antagonist of the pro-inflammatory interleukin IL-36 and it is secreted to prevent the development of an exacerbated inflammatory response to stressors (52). VEGF is a growth factor, and it possesses pro-angiogenic activity, promoting endothelial cell survival, cell migration and increasing vascular permeability (53). We observed no differences between infected and healthy animals, suggesting that they are unlikely to serve as diagnostic biomarkers for Brucella infection in buffaloes.

Finally, we aimed to identify the cytokine set which better discriminate Brucella-infected and healthy Mediterranean Buffaloes. Three canonical analyses were performed sequentially to reduce the number of cytokines while ensuring effective discrimination between the two groups of animals. From the initial 15, we narrowed it down to five: our analysis showed that the quantitative determination of IFN-*γ*, in parallel with the chemokine IP-10 and three pro-inflammatory cytokines (IL-6, IL-1α and IL-1β) could be useful in the diagnosis of brucellosis in Mediterranean Buffaloes. The infected group is characterized by high values of IFN-γ and IP-10, unlike the other three pro-inflammatory markers, which have higher values in healthy subjects.

Overall, our data suggest that IFN-γ, IP-10, IL-1a, IL-1ß, IL-6 could enhance brucellosis diagnosis in Mediterranean Buffaloes. These preliminary observation should be validated on a larger set of samples, in order to properly establish the sensitivity and specificity of these ELISAs. In particular, samples from buffaloes infected with other Gram-negative bacteria should be included in the analysis, to evaluate the specificity of these potential biomarkers and to implement the diagnosis of Brucella in this specie, in order to avoid un-necessary animal sacrifices. In conclusion, these preliminary findings provide a foundation for developing cytokine-based diagnostic tools for Brucella infection in Mediterranean Buffaloes.

## Data Availability

The raw data supporting the conclusions of this article will be made available by the authors, without undue reservation.
