# Supplementary material for: Exploring potential cytokine profiles as diagnostic biomarkers for brucellosis in Mediterranean Buffaloes
Source: Front Vet Sci. 2025 May 8;12:1583858. doi: 10.3389/fvets.2025.1583858 (PMC12097277; doi:10.3389/fvets.2025.1583858)
Supplement: Supplementary file 1 [file Table_1.docx]

**Table S1. Information of Mediterranean buffaloes enrolled in the study.**

|  | **Breeding locations** | **Brucella antibodies - RBT** | **Brucella antibodies -CTF** | **Brucella DNA -PCR** | **PCR^+^ ORGANS** |
| --- | --- | --- | --- | --- | --- |
| Healthy_1 | Salerno | NEG | N.P. | N.P. | - |
| Healthy_2 | Salerno | NEG | N.P. | N.P. | - |
| Healthy_3 | Salerno | NEG | N.P. | N.P. | - |
| Healthy_4 | Salerno | NEG | N.P. | N.P. | - |
| Healthy_5 | Salerno | NEG | N.P. | N.P. | - |
| Healthy_6 | Salerno | NEG | N.P. | N.P. | - |
| Healthy_7 | Salerno | NEG | N.P. | N.P. | - |
| Healthy_8 | Salerno | NEG | N.P. | N.P. | - |
| Healthy_9 | Salerno | NEG | N.P. | N.P. | - |
| Healthy_10 | Salerno | NEG | N.P. | N.P. | - |
| Healthy_11 | Salerno | NEG | N.P. | N.P. | - |
| Healthy_12 | Salerno | NEG | N.P. | N.P. | - |
| Healthy_13 | Salerno | NEG | N.P. | N.P. | - |
| Healthy_14 | Salerno | NEG | N.P. | N.P. | - |
| Healthy_15 | Salerno | NEG | N.P. | N.P. | - |
| Healthy_16 | Salerno | NEG | N.P. | N.P. | - |
| Healthy_17 | Salerno | NEG | N.P. | N.P. | - |
| Healthy_18 | Salerno | NEG | N.P. | N.P. | - |
| Infected_1 | Caserta | POS | 106.4 U.I./ml | POS | retropharyngeal lymph nodes |
| Infected_2 | Caserta | POS | 212.8 U.I./ml | POS | mandibular lymph nodes |
| Infected_3 | Caserta | POS | 212.8 U.I./ml | POS | uterus |
| Infected_4 | Caserta | POS | 26.6 U.I./ml | POS | mammary gland |
| Infected_5 | Caserta | POS | 26.6 U.I./ml | POS | supra-mammary lymph nodes |
| Infected_6 | Caserta | POS | 26.6 U.I./ml | POS | uterus |
| Infected_7 | Caserta | POS | 265.6 U.I./ml | POS | retropharyngeal lymph nodes |
| Infected_8 | Caserta | POS | 132.8 U.I./ml | POS | uterus |
| Infected_9 | Caserta | POS | 33.2 U.I./ml | POS | mandibular lymph nodes, uterous |
| Infected_10 | Caserta | POS | 80 U.I./ml | POS | mammary gland |
| Infected_11 | Caserta | POS | 66.4 U.I./ml | POS | mammary gland |
| Infected_12 | Caserta | POS | 80 U.I./ml | POS | uterus |
| Infected_13 | Caserta | POS | 320 U.I./ml | POS | retropharyngeal lymph nodes, iliac lymph nodes, uterus |
| Infected_14 | Caserta | POS | 320 U.I./ml | POS | supra-mammary lymph nodes, mandibular lymph nodes, spleen |
| Infected_15 | Caserta | POS | 320 U.I./ml | POS | retropharyngeal lymph nodes, mandibular lymph nodes, iliac lymph nodes, uterus |
| Infected_16 | Caserta | POS | 320 U.I./ml | POS | retropharyngeal lymph nodes |
| Infected_17 | Caserta | POS | 320 U.I./ml | POS | retropharyngeal lymph nodes, mandibular lymph nodes, iliac lymph nodes |
| Infected_18 | Caserta | POS | 320 U.I./ml | POS | uterus |
| Infected_19 | Caserta | POS | 320 U.I./ml | POS | mandibular lymph nodes |
| Infected_20 | Caserta | POS | 212.8 U.I./ml | POS | mammary gland |

N.P. Not performed.
